# High-Velocity Impact of Polymer Aerosol Particles on Soft Substrates: Experiments and Simulations

**DOI:** 10.1021/acs.langmuir.5c03939

**Published:** 2025-12-11

**Authors:** Marc C. Thiel, Hongyu Gao, Matthias B. B. Brzoska, Lukas Siegwardt, Markus Gallei, Martin H. Müser, Karen Lienkamp

**Affiliations:** † Chair of Polymer Materials, Campus C 4.2, 9379 Saarland University, 66123 Saarbrücken, Germany; ‡ Chair of Materials Simulation, Campus C 6.3, 9379 Saarland University, 66123 Saarbrücken, Germany; ¶ Chair of Polymer Chemistry, Campus C 4.2, 9379 Saarland University, 66123 Saarbrücken, Germany; § Saarene, Saarland Center for Energy Materials and Sustainability, Campus C 4.2, 66123 Saarbrücken, Germany

## Abstract

We study the high-velocity impact of spherical polystyrene (PS) particles on polymer substrates to gain insight into the initial stages of powder aerosol deposition (PAD), a sustainable, solvent-free technique for polymer and ceramic thin film deposition with promising application potential for single functional or multilayered, multimaterial coatings. Single-particle impacts were investigated experimentally using a PAD setup and compared to molecular dynamics simulations, in which the particle diameter and impact velocity were systematically varied. The simulated particle shapes show good agreement with those observed experimentally via atomic force microscopy. After impact, the initially spherical particles deform into shapes resembling cylindrical domes, similar to those known from the impact of yield-stress fluids. Scaling behavior extracted from the simulations provides estimates of the otherwise not directly measurable experimental impact velocities and reveals key aspects of the particles’ deformation mechanism during impact, which is driven by a temperature increase causing viscoplastic flow. Our results suggest that both adhesion and deformation of PS on polymer substrates during PAD are primarily governed by viscoplastic deformation rather than by fragmentation as typically observed in ceramic systems, or jetting due to adiabatic shear instabilities, as found in the closely related cold spray process. The insights gained in our study suggest that efficient PAD of polymers is easier for materials with good plastic deformability and thereby contribute to identifying material properties and design principles for future polymer PAD systems.

## Graphical abstract

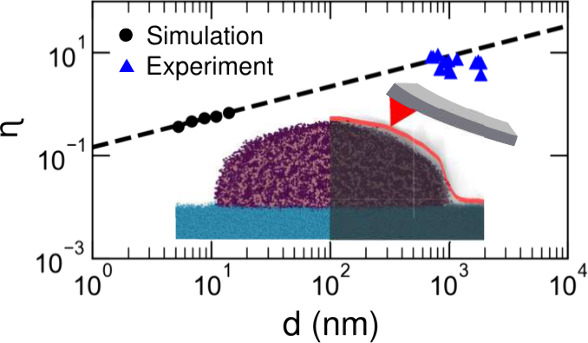

## Introduction

Powder aerosol deposition (PAD) is a process originally developed to obtain thin layers of ceramics without the need for high-temperature sintering. In a typical PAD experiment, micrometer-sized inorganic particles, e.g., alumina or titanium dioxide, are accelerated to supersonic speed. When hitting the target substrate (typically a hard substrate such as steel), these particles undergo plastic deformation and break into nanosized fragments, leading to the formation of a dense, nanocrystalline ceramic film. This mechanism is referred to room-temperature impact consolidation.^1–3^

Despite the well-established use of PAD for ceramics, its application to polymers has so far remained limited.^4^ This is due to the availability of numerous simple methods for producing polymer films, including solution-based techniques (e.g., spin coating or dip coating) and melt-based approaches (e.g., melt blowing, calendaring and hot pressing). However, these techniques are limited to soluble, thermoplastic polymers. In contrast, PAD may offer a viable route for producing robust coatings with strong adhesion, particularly for functional polymers with limited solubility. The process most closely related to PAD for polymers is Cold Spray (CS), where polymer coatings are obtained by spraying particles at temperatures of 200–500 °C onto substrates using a carrier gas overpressure. CS of polymers is comparatively better understood but differs from PAD in important parameters such as particle size, particle impact velocity, and carrier-gas temperature:^5^ Particles used in CS are larger (tens of microns), faster and experience higher temperatures compared to PAD, which is a room temperature process. The dynamics and mechanism of polymer deposition in PAD should, therefore, differ substantially from those of polymer CS.

Deposition of ceramic particles in PAD has been investigated both experimentally and theoretically in great detail.^1–3,6–8^ However, since the thermal and mechanical properties of polymers are substantially different from those of ceramics, these results are not directly transferable, and mechanistic studies of polymers in PAD remain scarce.^9–11^ Since the dynamics and mechanism of polymer deposition in PAD differ from those of ceramic PAD, differences in the resulting microstructure - and thus in film properties - are expected. To our knowledge, there is so far only one case in which PAD was used successfully to prepare a pure polymer film.^11^ In that study, polyimide (PI) powder was deposited onto a gold-coated glass substrate to form a dielectric layer, resulting in a 19 μm thick, rough PI film that was described as dense and free of major defects.^11^ SEM images indicated that film formation occurred mainly through plastic deformation of primary particles rather than fragmentation,^11^ and no further details on the deposition mechanism were provided.^11^ This highlights the need to gain further insight into the PAD process for polymers. When more fully understood, PAD could not only become an interesting technique for the processing of poorly soluble polymers, but also a unique avenue to obtain multilayered polymer–ceramic composites.

The underlying process of a high-velocity impact of a polymer droplet (below its glass transition temperature) on a substrate also appears to be unexplored from a theoretical perspective. Even classifying the process is challenging, as the droplet can undergo brief but significant heating during the collision. As a result, the droplet, and potentially a part of the polymeric substrate may transition from an initially brittle plastic to a viscoelastic, shear-thinning fluid during the collision, and return into a glassy state afterward. Passing through a viscoelastic state would allow energy dissipation, increase the material’s toughness and help to counteract particle fracture. This may be a key reason why polymer particles during PAD do not fragment easily, in contrast to ceramics, and abstain from forming the jet-like structures observed during CS.^12–14^

In light of the above-mentioned applications and open scientific questions, this work aims to study the initial step of film formation in an all-polymer PAD system. Specifically, we examine the impact of a polymer particle on a pristine region of a soft substrate experimentally and *in silico*. To this end, PS particles with an average diameter of about 1.15 μm were accelerated onto a polystyrene substrate and subsequently analyzed by atomic force microscopy (AFM). The results were compared to molecular dynamics (MD) simulations. While the MD model used smaller particles than those in the experiments, the implications of this scale difference were shown to be minor, so that the combined results of the laboratory and MD experiments give further insight into the adhesion and deformation mechanisms in polymer PAD.

## Materials and Methods

### Preparation of the PS Powder

#### Materials

Styrene (S, 99%) was purchased from Fisher Scientific (Fisher Scientific GmbH, Schwerte, Germany). Sodium persulfate (NaPS ≥ 98%) was purchased from Sigma-Aldrich (Merck KGaA, Darmstadt, Germany). Sodium chloride (NaCl, analytical grade, 99.5%) was purchased from Grüssing (Grüssing GmbH, Filsum, Germany).

#### Synthesis

The PS particles used for the single-impact tests were synthesized by surfactant-free emulsion polymerization. Prior to polymerization, radical inhibitors were removed by passing S monomer through a basic alumina column (50–200 μm, Acros Organics). Polymerization was conducted in a 1 L three-necked round-bottom flask equipped with a reflux condenser and a mechanical stirrer with a crescent-shaped PTFE stirring rotor under a nitrogen atmosphere. The flask was filled with 615 g deionized water and 0.58 g NaCl. For degassing, the batch was stirred for 15 min at 230 rpm at room temperature. Afterward, 65.45 g S monomer was added dropwise. 75 g of deionized and degassed water was used for rinsing and thereby added to the reaction mixture. Under constant stirring at 230 rpm, the emulsion was heated up to 70 °C in an oil bath. Simultaneously, 0.54 g NaPS initiator was dissolved in 10 g deionized and degassed water. Under constant stirring at 230 rpm, the NaPS solution was added to the preheated emulsion to initiate the polymerization. Twenty gram of deionized and degassed water was used for rinsing and thereby added to the reaction mixture. After 24 h, the reaction mixture was cooled to room temperature, filtered through a 125 μm nylon sieve, and subsequently freeze-dried to obtain the PS powder.

#### Size Exclusion Chromatography (SEC)

The molecular mass distribution of the linear PS chains forming the PS particles was analyzed by SEC using a 1260 Infinity II system (Agilent Technologies, Santa Clara, CA, USA). Tetrahydrofuran was used as the mobile phase (HPLC grade, flow rate 1 mL min/1) on an SDV column set (SDV precolumn; SDV 5 μm 103 Å; SDV 5 μm 105 Å; SDV 5 μm 106 Å) from Polymer Standard Services (PSS, Mainz, Germany) with a PSS SECurity2 UV detector. Calibration was carried out using PS standards from PSS. PSS WinGPC UniChrom V 8.31 was used for data acquisition and evaluation of the measurements. The number-average molecular mass*M*_*n*_ determined was 59.200 g/mol and the weight-average molecular mass *M*_*w*_ was 299.100 g/mol, corresponding to a polydispersity of 5.05.

#### Particle Size Distribution

The hydrodynamic diameter of the particles was determined by dynamic light scattering (DLS) using a Zetasizer ZS 90 (Malvern Instruments Ltd., Worcestershire, UK) equipped with a 4 mW, 633 nm He–Ne laser at an angle of 90°, with a 5-fold determination of 15 runs. The particle size distribution after polymerization is shown in Figure 1a. The mean hydrodynamic diameter was 1.15 μm.

**Figure 1:**
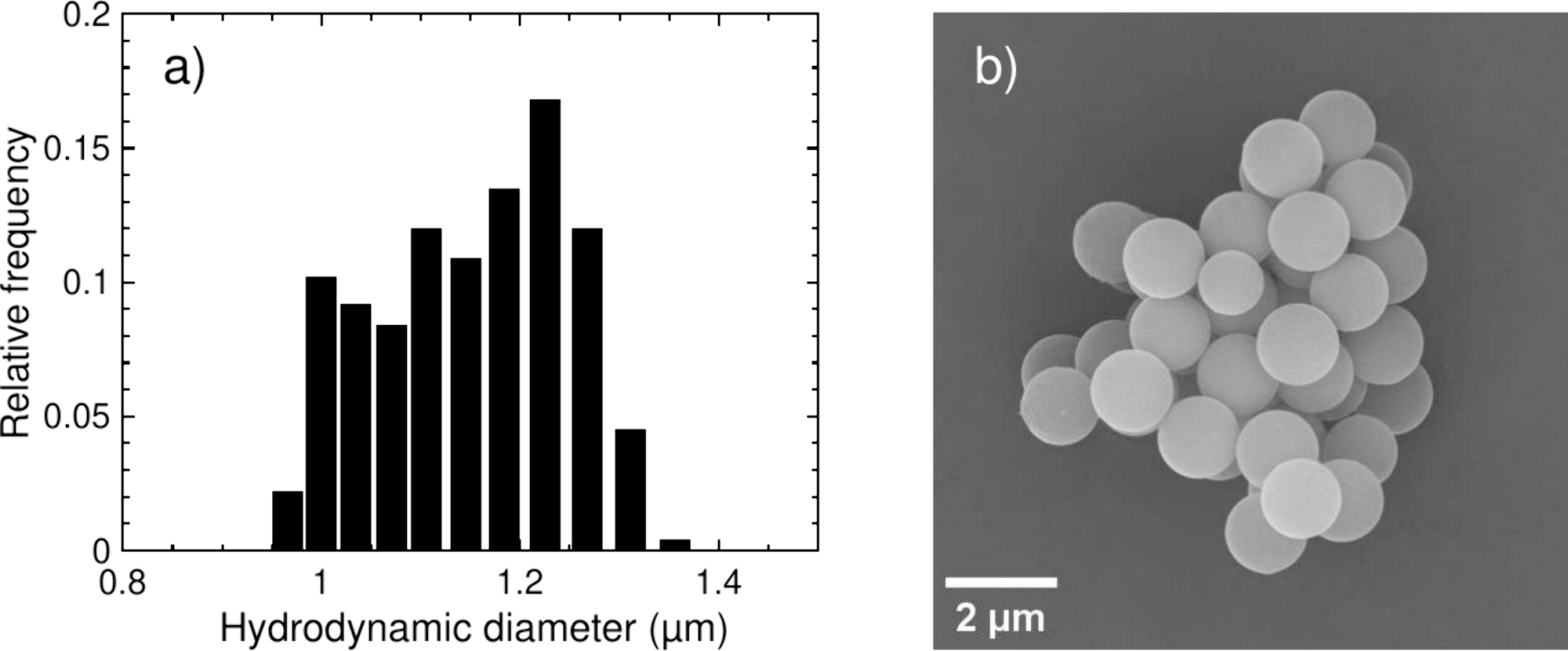
(a) Size distribution of the PS particles obtained by DLS. (b) SEM image of representative particles after freeze-drying.

#### Scanning Electron Microscopy (SEM)

The particle morphology was studied using a Zeiss Sigma 300 VP scanning electron microscope (Zeiss, Oberkochen, Germany). For this purpose, a few milligrams of the freeze-dried powder were distributed on a double-sided adhesive conductive tab strip (Plano GmBH, Wetzlar, Germany). The sample was then sputtered with gold using a Cressington Sputter Coater 108 Auto (Tescan GmbH, Dortmund, Germany; process parameters: 20 mA, 0.1 mbar, 45 s). A SEM image taken from representative particles at 20 kV is shown in Figure 1b.

### Single-Impact Experiments

#### Powder Aerosol Deposition

Commercially available PS plates (KTK Kunststofftechnik, Germering, Germany) were used as substrates. The plates were cut into 15 mm × 15 mm × 1 mm squares, cleaned in an ultrasonic bath, rinsed with ethanol, and dried at room temperature. The basic setup of the PAD device used for the single-impact tests is described elsewhere.^2^ In short, it consists of two chambers: an aerosol chamber containing the particle powder, and an evacuated chamber with a movable sample holder onto which the substrate is mounted. Using nitrogen as a carrier gas, the polymer particles were aerosolized by a vibratory plate and accelerated onto the substrate through a nozzle, thus reaching high impact velocities when hitting the PS substrate, which was moved at 100 mm/s over the nozzle. Table 1 lists the deposition parameters used. The gas flow was measured in standard liters per minute (slm). To ensure that single particles hit the substrate without colliding with subsequent particles, only one crossing of the substrate across the nozzle with a very high scan rate was performed (1 scan = 2 crossings). After deposition, the substrate was cleaned in an ultrasonic bath to remove loose particles, rinsed with ethanol, and dried at room temperature.

**Table 1:** PAD Parameters for the Single-Impact Experiments^a^

Parameter	Value
Nozzle type	Convergent slit nozzle
Orifice size of nozzle	10 × 0.5 mm
Distance nozzle–substrate	10 mm
Gas flow rate/gas type	3 slm/N_2_
Pressure in vacuum chamber	5.9 × 10^–1^ mbar
Pressure in aerosol chamber	98 mbar
Scan rate	100 mm/s
Vibration frequency	400 rpm

a slm = standard liters per minute.

#### Atomic Force Microscopy (AFM)

The topography of the substrate surface after deposition was imaged by a Dimension Icon atomic force microscope (Bruker, Billerica, MA, US) in ScanAsyst mode using a ScanAsyst-Air cantilever (Bruker, Camarillo, CA, US) with a resonance frequency of 70 kHz and a spring constant of k = 0.4 N/m. To analyze the impacted single particles, a predefined area of 40 × 40 μm was mapped out on the substrate before and after deposition. The particles found in this field were then scanned individually with an imagesize of 5 × 5 μm. All AFM images were recorded with a scan rate of 1 Hz and consisted of 512 × 512 pixels. All AFM images were processed using Gwyddion 2.66. First, the images were leveled by subtracting the mean plane. Some images were then subjected to light median filtering (window size up to 5 pixels) to reduce measurement artifacts. The height sensor data were subsequently exported as ASCII data matrices and further analyzed in MATLAB R 2024b. To isolate individual particles, a region of interest (ROI) was defined based on a moving mean filter. The algorithm scanned the image using fields of 64 × 64 pixels with 50% overlap. Fields in which more than 20% of the height values exceeded an upper threshold and less than 20% fell below a lower threshold were marked as containing a prominent feature. The outermost regions of these marked fields defined the boundaries of the ROI. The *x*–*y*–*z* data within the ROI were converted to polar coordinates centered at the particle’s centroid. The average radial height profile and the corresponding normalized probability density were computed using concentric circles extending from the particle center to the ROI boundary. Based on this probability density, each data point was classified as either belonging to the particle or to the substrate, depending on whether its local value exceeded a defined threshold of 0.1. The mean substrate level was subtracted from all points. From the corrected particle data, the center of mass, in-plane and out-of-plane radii of gyration, average height, lateral extent, and deformed volume were calculated. The flattening index, defined further below in eq 1 was then computed from the ratio of in-plane to out-of-plane radii of gyration, while the initial particle diameter was back-calculated from the deformed volume, assuming volume conservation and an initially spherical shape, which appears plausible based on Figure 1b. The flattening index as a function of initial radius was subsequently compared to simulation data.

### Simulation

Molecular dynamics (MD) simulations were performed using the LAMMPS^15^ software package to investigate the collision dynamics between a polystyrene (PS) nanoparticle and a PS substrate. The PS molecules were modeled using the MARTINI coarse-grained (CG) force field,^16^ where each styrene repeat units was represented by four beads - three corresponding to the phenyl group, and one to the aliphatic backbone. Bonding interactions (bond stretching and angle torsion) and van der Waals interactions were described using parameters from ref 17. The MARTINI model was selected over other candidates, e.g., the Kremer-Grest (KG) model,^18^ due to its preparameterized representation of PS, which offers a practical balance between computational efficiency and physicochemical fidelity. While the MARTINI model has imitations in capturing absolute quantitative accuracy, it reliably reproduces essential polymer properties relevant to collision phenomena and eliminates the need for *ad hoc* force-field parametrization, particularly for temperature-dependent behavior.

The simulated system comprised two primary components: a spherical PS nanoparticle (“ball”) and a nominally flat PS substrate. The PS ball consisted of linear chains with 100 styrene repeat units each, and its diameter ranged from 5.3 to 14.0 nm, corresponding to 5 to 80 polymer chains. While these diameters are smaller than those typically used in experimental studies, they were carefully selected to retain physical relevance to real-world collision behavior. By matching key morphological outcomes (e.g., postimpact height-to-width ratios) between simulations and experiments, we make sure that our scaled-down system faithfully represents the underlying collision physics while remaining computationally tractable. The PS substrate was constructed as a 34.6 × 34.8 × 5.0 nm^3^ slab composed of identical PS chains. To avoid artifacts from substrate deformation or artificial stiffness, a rigid gold (111) layer was placed beneath the polymer slab and interacted with the PS chains via the same nonbonding interactions. Periodic boundary conditions were applied in the in-plane (*xy*) directions to minimize edge effects.

Prior to the collision simulations, both the PS ball and substrate were thoroughly equilibrated to ensure structural and thermodynamic stability. The system was initially heated to an elevated temperature to accelerate chain relaxation, then cooled at a rate of 10 K/ns to the target temperature of 300 K. The resulting average densities of the PS ball and substrate were approximately 1.03 g/cm^3^, closely matching the experimental value of 1.054 g/cm^3^.^19^ A subsequent equilibration phase of at least 200 ns was added to reach a near-equilibrium state. Throughout the simulations, the substrate temperature was maintained at 300 K using a Langevin thermostat, which was applied to the bottom 10% of the substrate. The PS ball was similarly thermostated at 300 K during initial equilibration and ballistic flight. Just prior to impact, the thermostat was removed to allow an undisturbed collision. Following contact, the ball was no longer thermostated, and its temperature was allowed to rise due to the conversion of kinetic energy into internal energy during impact. The substrate remained thermostated at 300 K, serving as a thermal reservoir for postcollision equilibration.

Collision simulations were performed by launching the PS ball normal to the substrate at velocities ranging from 100 to 1,260 m/s. This range was chosen to span deformation regimes from quasi-static contact to near-fragmentation, allowing comprehensive investigation of impact dynamics. While the exact velocities of PS nanoparticles in the corresponding experiments cannot be easily quantified, the selected values in the simulations facilitate exploration of fundamental collision mechanisms across relevant regimes. To reduce the influence of initial structural asymmetries in the PS ball, each simulation was repeated with the ball rotated 90° about the *x*- or *y*-axis, and the results were averaged across these orientations to enhance statistical robustness. Following impact, the system was equilibrated for an additional 2 ns to allow structural relaxation, during which only minimal changes were observed, supporting reliable postcollision analysis. The time step was 2 fs and the damping time constant was 200 fs throughout all the simulations.

## Results and Discussion

The ballistic experiments, involving the acceleration of PS particles onto a PS substrate, were performed in the PAD setup. We chose operation parameters that resulted in the impact of individual PS particles onto the PS substrate without being hit by successive particles. For this, we kept the scan rate during PAD sufficiently high, so that any two particles were unlikely to impact the substrate at the same location. The thus treated substrate was imaged by AFM as described in the Methods section. Figure 2 shows an AFM height sensor image of a specific substrate position before and after the PAD process. It reveals that most impacted particles were indeed well isolated from each other. Particles were first identified in an 40 × 40 μm^2^ overview scan and then imaged at higher resolution in a 5 × 5 μm^2^ scan field. Several such overview scans were performed to obtain robust statistics. The height sensor data were processed as described in the Methods section to obtain a radial height profile. From that profile, points corresponding to the particle and the substrate, respectively, were separated and corrected to the substrate level. The particle diameter, height, center-of-mass, radius of gyration, and volume were then determined. The shapes of several particles were analyzed, and three representative examples are shown in Figure 3 as top view, 3D rendering, and average radial height profile, with particle 1 corresponding to the example shown in Figure 2 and particles 2 and 3 obtained from other regions of the substrate following the same procedure.

**Figure 2:**
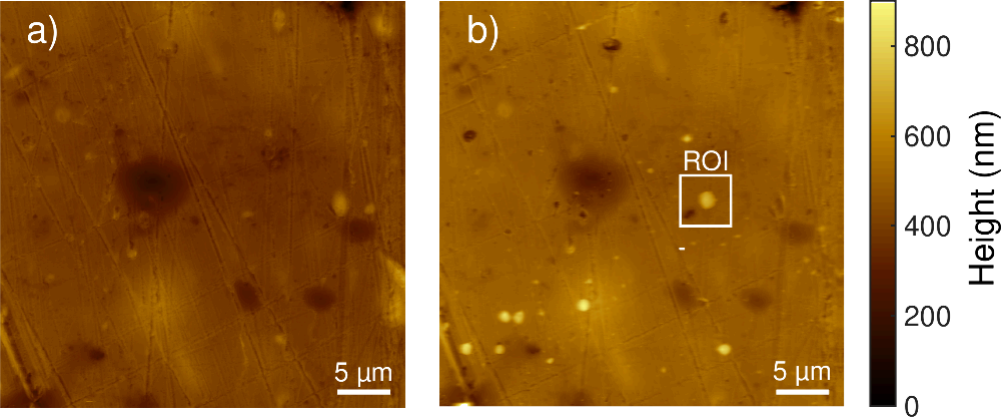
AFM height sensor images of the PS substrate (a) before PAD and (b) after PAD with 1 crossing of the substrate at a scan velocity of 100 mm/s. The particle in the region of interest is one of the particles that is focused on in the following (particle 1, Figure 3).

**Figure 3:**
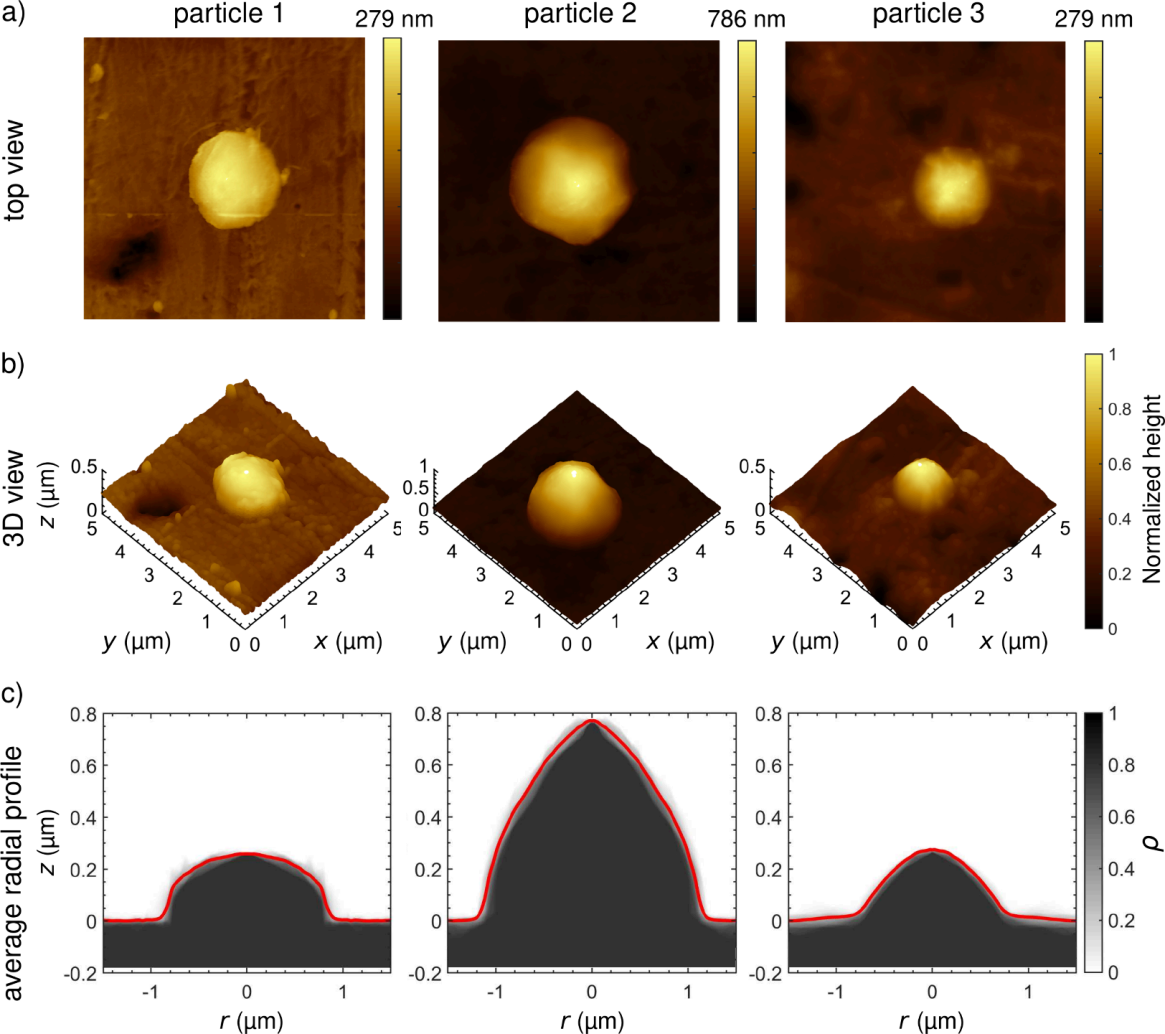
(a) Top view, (b) 3D rendering, and (c) average radial profile of three representative particles after deposition. Particle 1 is the one highlighted in Figure 2. The cross sections in panel c are based on the normalized probability density of heights ρ (grayscale) derived from AFM data of the particle within the ROI and the resulting average radial profile of the particles (red line).

Most particles retain a quasi-circular shape parallel to the *xy*-plane, while they adopt a dome-like shape perpendicular to the *xy*-plane, like particle 1 shown in Figure 3a. The observed shape is similar to that of yield-stress fluids.^20,21^ In these, the onset of flow is governed by the shear stress (more precisely by the second invariant of the deviatoric stress tensor, which is conceptually analogous to the von Mises yield criterion used in plasticity theory). Agreement between the here observed final shapes and that of yield-stress fluids appears to be largest in cases where the yield-stress fluid droplets are still in viscous flow.

In rare occasions, the shape of our particles show signs of a conical protrusion, however, much less prominently than those displayed by yield-stress fluids.^20^ The deformed particles also resemble the shapes of polymer particles observed after deposition by CS processes, both in experimental^14,22–26^ and simulation studies,^14,25–27^ although the initial particle diameters in these studies were approximately 1 order of magnitude larger than in our experiments, and the process parameters differed in several important aspects. Among these studies, the deformed particle morphologies reported in Duran et al.^26^ show the closest resemblance to our results, a correspondence that will be discussed in more detail further below.

In the MD simulations, the deposited particles also adopt shapes that are close to circular in cross sections parallel to the *xy*-plane axis, and dome-like perpendicular in the plane containing the *z*-axis and the droplet’s center of mass. The final aspect ratio of the largest particle deposited at the highest velocity remains somewhat below the ones typically found experimentally, which is likely due to the size difference between *in-silico* and PAD laboratory experiments. This discrepancy will be investigated further below.

Molecular snapshots taken before, during, and after the deposition process are shown in Figure 4. They reveal that impacting particles are still far from fully flattened when the force between the deposited particle and substrate polymers reaches its maximum (Figure 4b). Thus, the particles remain in substantial viscous and/or plastic flow after the impact force has peaked. The ultimate shape of the simulated particle in Figure 4(d) is affinely equivalent to the experimental cross sections shown in Figure 4(e). In other words, the two shapes coincide when independent scaling factors are applied along the normal and in-plane directions.

**Figure 4:**
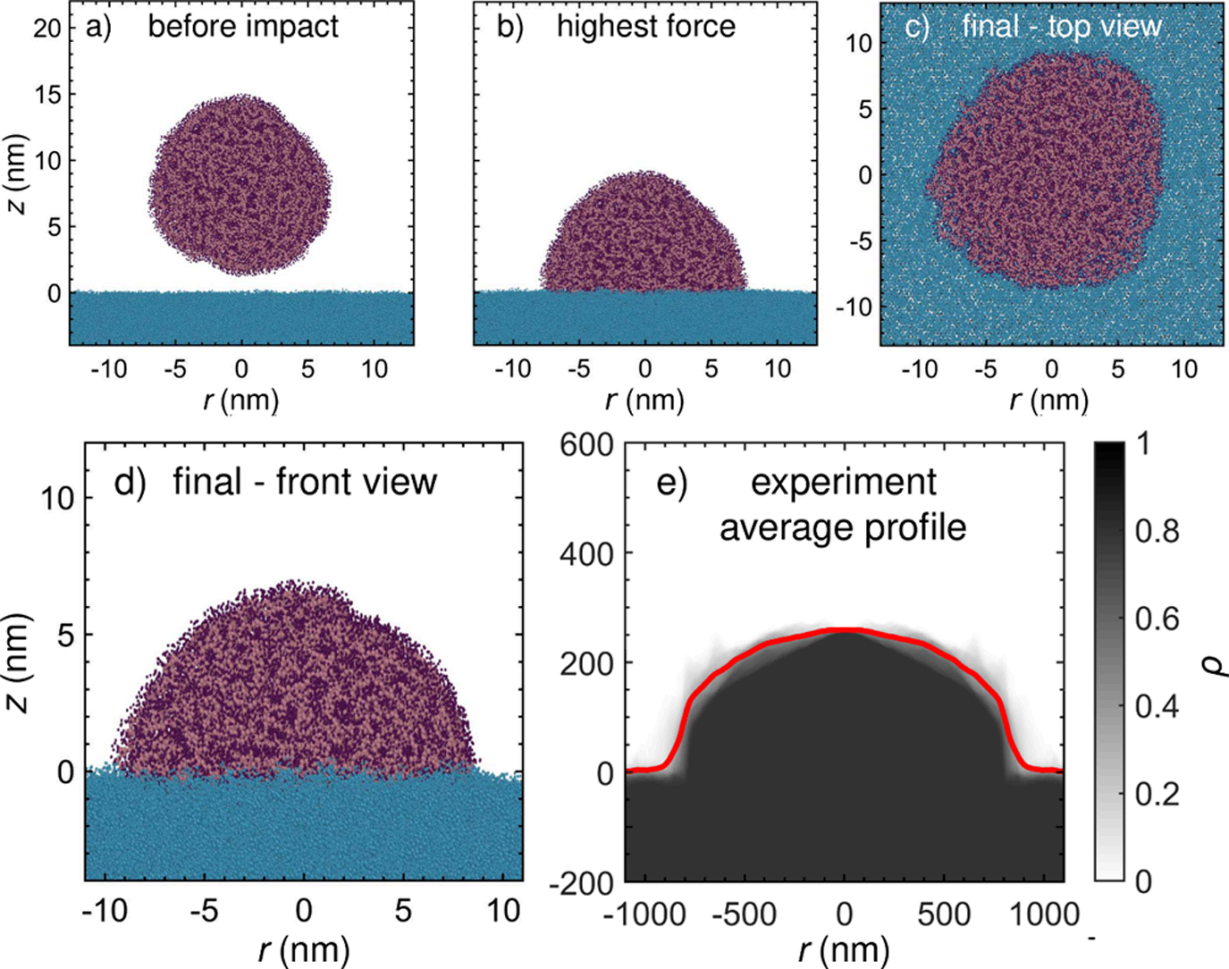
MD snapshots of a PS nanoparticle (mean diameter 14 nm) (a) before impact, (b) at the moment of maximum force exerted by the particle on the substrate (both shown as cross sections), and in the final state: (c) top view and (d) cross-section. e) Average particle profile (red line) and normalized probability density of heights ρ (grayscale) derived from AFM data of the particle within the ROI. A corresponding movie showing the relative atomic displacements with respect to the particle’s center of mass during the simulated impact process is provided as Supporting Information.

It is far from trivial that the simulated and experimental particle shapes look similar, given the vast number of possible outcomes when solidified droplets impact a surface. In addition to the scale difference between the simulated nanometer-scale particles and the experimentally used micrometer-sized particles, key differences exist in how mechanical energy is converted and thermal behavior is represented. One important difference is that the redistribution of the center-of-mass kinetic energy into localized, quasi-thermal modes (bond-stretching, torsional motion, center-of-mass, etc.) is not accurately reproduced in a classical MD simulation. On the one hand, the model has fewer degrees of freedom, which makes its specific heat capacity artificially small, so that temperature increases are overestimated. On the other hand, the simulated degrees of freedom are classical, which has the opposite effect. This issue turns the calculation of the specific heat capacity - and thus the prediction of a temperature increase with classical MD - into a nontrivial exercise even in thermal equilibrium,^28^ and is supposedly at the root of why even carefully conducted all-atom polymer simulations overestimate thermal conductivity.^29,30^

Other differences between experiment and simulation relate, for example, to the ratio of impact duration and the time it takes for heat to flow away from the deposited particle. The number of entanglements (normalized either per unit volume or to the entire droplet) certainly also differs between simulations and experiments, though it is not clear to what extent they matter in our case. Nonetheless, scaling laws and dynamics are often universal in soft-matter systems,^31^ and similar dynamics can be observed when the governing dimensionless numbers match. In this context, a number and that is particularly relevant for the impact dynamics of droplets on surfaces - and likely to be well reproduced in simulations - is the plastic number *Pl* = σ_*y*_/σ_*k*_. It is defined as the ratio of the yield stress, σ_*y*_, which classical simulations can reproduce reasonably well, to the kinetic stress, σ_*k*_, representing the kinetic energy tensor of the center of mass per volume. Thus, the good agreement between experiment and simulations is supposedly not fortuitous, so that useful information can be gained by further analyzing the MD simulations.

To better relate the nanometer-scale simulations to the micrometer-scale experiments, one needs to understand how the final shape of the deposited particles depends on their initial diameter *d* and on their initial velocity *v*. Since velocities could not be measured in our PAD setup, we analyzed these dependencies using molecular dynamics, as shown in Figure 5. The final shape of the particles are characterized by the gyration-tensor elements, which we reduce to an in-plane and an out-of-plane radius of gyration *R*_*g*,*in*_ and *R*_*g*,*out*_. Their ratio is unity in equilibrium, so that we define and study a flattening index instead,

1 $$\eta =\frac{R_{g,in}}{R_{g,out}}-1$$

see Figure 5. We note that 1 + η corresponds to the width to height ratio of the MD snapshots to within clearly less than 5%.

**Figure 5:**
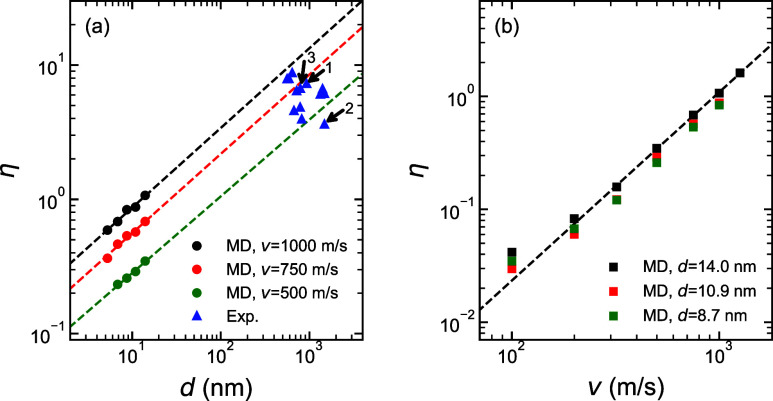
Flattening index η as functions of (a) the initial particle diameter *d* and (b) the impact velocity *v*. Dashed lines show power-law fits to the MD results, yielding exponents of (a) 0.59 (black), 0.59 (red), 0.57 (green), and (b) 1.67. The simulation data shown in panel a, which were obtained for the velocities of *v* = 1, 000 m/s (black circles), 750 m/s (red circles), and 500 m/s (green circles), respectively, is complemented by experimental data (blue triangles) for particles impacting the surface with an unknown velocity. The numbers in panel a relate to the particle numbers shown in Figure 3.

At a fixed impact velocity of *v* = 500 m/s, *v* = 750 m/s, or *v* = 1,000 m/s, evidence for a scaling close to η ∝ *d* ^3/5^ is revealed in Figure 5a. Although the validity of the power-law scaling above 100 nm remains uncertain, the agreement between experiment and simulation is reassuring. (Increasing *d* is very challenging as doubling the diameter implies eight-times the computational burden.) Power law scaling is also observed from the simulations for the η­(*v*) dependence, at least for velocities exceeding 200 m/s, where a crossover of scaling laws seems to occur, see Figure 5b. At that velocity, the (negative) *zz*-component of the kinetic stress is σ_*k*_ ≈ 1 g/cm^3^× (250 m/s)^2^ ≈ 60 MPa. This is rather close to the yield strength of room-temperature PS, σ_*y*_ ≈ 50 MPa. Given the short duration of impact, which is merely of the order of 10 ps in the simulations, it is clear that (plastic) flow occurs during the initial phases of impact prior to heating but that flow during the later stages is likely to benefit from heating effects.

To characterize the impact dynamics in more detail, Figure 6 shows (a) the normal force, (b) the potential energy, (c) the center of mass position, (d) the flattening index, (e) the effective temperature increase over time, and (f) the density contours at selected time points. The normal force - defined as the sum of forces in *z*-direction on atoms initially belonging to the droplet - peaks roughly at 6 × 10^–3^ ns after impact for the highest velocity (1,000 m/s), and around 20 × 10^–3^ ns for the lowest one (100 m/s) (Figure 6a). These times correspond closely to the droplet diameter divided by the longitudinal and transverse sound velocities of polystyrene at ambient conditions, approximately *v*_l_ ≈ 2,400 m/s and *v*_t_ ≈ 1200 m/s, respectively. Upon contact, kinetic energy is rapidly converted into potential energy and reaches its peak around 10^–2^ ns for a velocity of 1000 m/s (Figure 6b), which is also the point where the force curve comes back to zero, and the position of the center of mass of the particle reaches its minimum (Figure 6c). The maximum of the flattening index η is reached a little later (Figure 6d). This delay corresponds to the time it takes for the particle to deform under the experienced stress. The effective temperature - defined via the kinetic energy of droplet atoms relative to the center-of-mass motion - peaks around 7 × 10^–3^ ns (Figure 6d), i.e. between the force maximum and the maximum flattening. This means that the temperature increase facilitates the viscoplastic flow induced by the impact stress. Cooling is faster for more strongly deformed droplets (e.g., at *v* = 1,000 m/s) than for less squished ones (e.g., *v* = 500 m/s), as the characteristic heat diffusion time into the substrate, τ ≈ *h*/*D*_*T*_, decreases with height. Typical values are *h* = 5 nm and *D*_*T*_ ≈ 1 · 10^–7^ m^2^/s, yielding τ ≈ 0.25 ns. The center-of-mass rebounds are surprisingly small in the simulations, possibly due to the temperature increase and resulting plastic flow. This aligns with the observation that the 100 m/s droplet, which heats only modestly, rebounds more than the one impacting at 1,000 m/s. In experiments, stronger rebounds are expected, as the vibrational quality factor scales with wavelength and thus with particle size.

**Figure 6:**
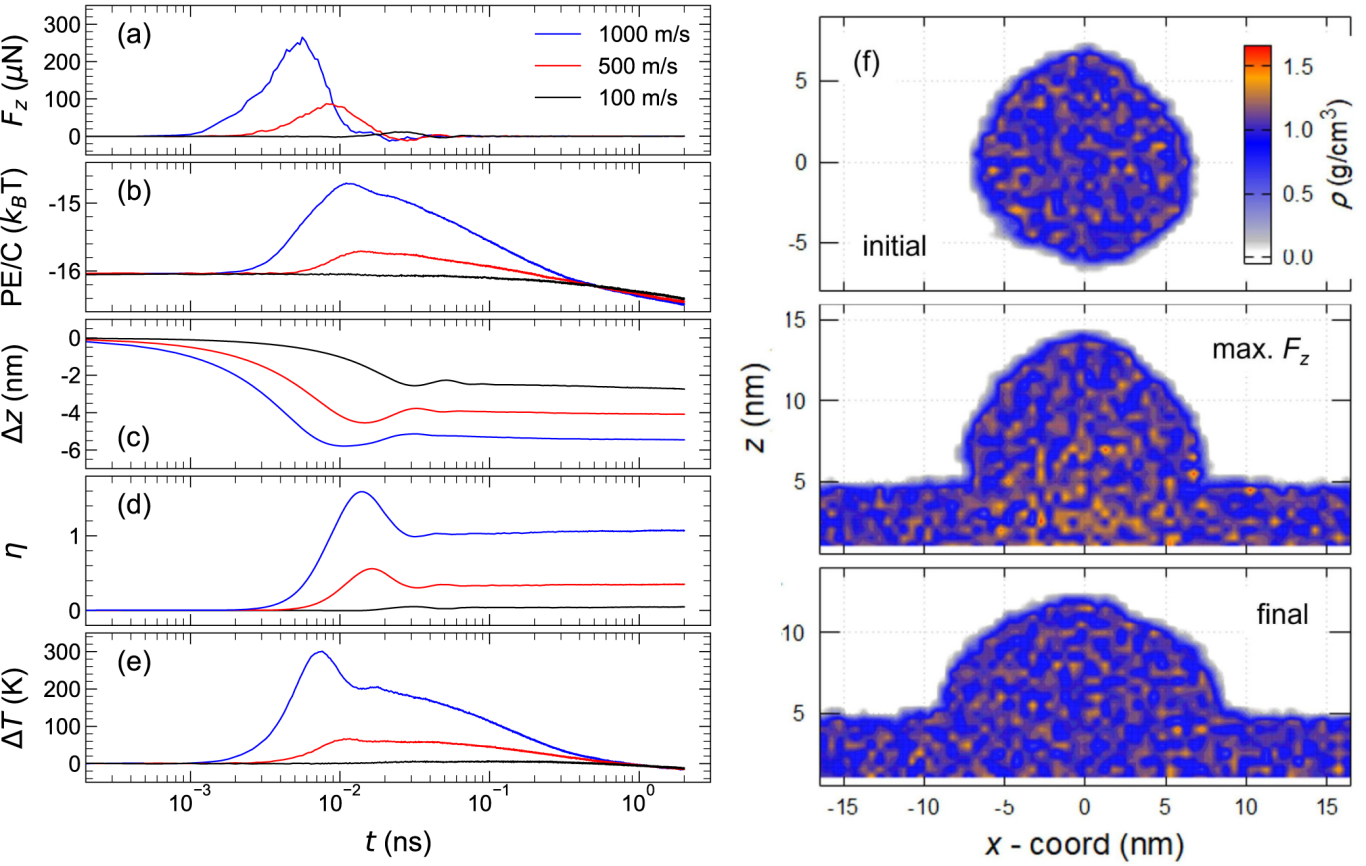
(a) Normal force, (b) potential energy per carbon atom (in units of *k*_*B*_ *T*), (c) center of mass shift after initial contact, (d) flattening index η, and (e) effective temperature increase as a function of time, where *t* = 0 at the moment of contact. (f) Density contours of the system at the initial, maximum *F*_*z*_, and final stages.

An anisotropy index was also evaluated for the tensor of gyration of individual polymers and averaged over the impacting particle to yield η_g_. Its value generally turned out slightly lower than η_p_ - for example, η_g_ = 0.81 vs η_p_ = 1.07 for *v* = 1,000 m/s and *d* = 14.0 nm. This suggests that shear-induced reorientation serves as the microscopic carrier of plasticity, enabling permanent shape change without covalent bond scission or full disentanglement. These findings imply that even moderately long chains - as used in our simulations - can exhibit plastic deformation mechanisms akin to those in experimental systems with much longer chains, provided they exceed a minimal threshold in chain length or entanglement density. We believe this to be the case because the scaling of η_p_ changes slope when the kinetic stress becomes comparable to the yield strength of polystyrene under laboratory conditions. The agreement of the simulations with the experiment should therefore not merely result from fortuitous error cancellation, but rather from capturing the correct underlying physics of local yielding. Nonetheless, the good agreement between simulations and experiments may well be aided by the fact that classical coarse-grained models of polymers have a specific heat capacity similar to that of real polymers.^28^ Furthermore, the relatively short chain length in our model (100 repeat units, corresponding to a number average molecular mass of 10,400 g/mol) is considerably lower than those measured experimentally, resulting in reduced entanglement and allowing molecules to rearrange more readily under impact-induced particle deformation.

Figure 6f shows snapshots of density contours for a particle before impact, at maximum force, and in the final state. The data shows that the overall density is uniform before impact and in the final state. At maximum force, there is a substantial density increase near the former particle-substrate interface. This density gradient is dissipated by the plastic flow of the particle. In addition, analysis of the impact shown in the movie (Supporting Information) reveals a substantial amount of mixing and thus adhesion between particle and substrate, which would not have occurred during a time as short as the impact duration if the system had been left in thermal equilibrium at ambient conditions. The mixing thus occurred due to the local heating during impact, supposedly in a similar fashion as argued to happen during the cold-spray of polymers on polymeric surfaces.^32^

In summary, the data suggests that the sequence of events of the impact are maximum force, followed by a maximum temperature increase, then a simultaneous maximum in the potential energy and a minimum in Δ*z*, followed by particle deformation. This is a clear indication for the contribution of the temperature increase to the particle flow. It is instructive to compare these results to experimental and simulation-based studies in the CS literature. These consistently show that the deformation behavior upon particle impact strongly depends on the nature of the polymer, particularly on its glass transition temperature *T*_*G*_, the molecular mass, and on the mechanical properties of the substrate.^14,22–26,33,34^ Process parameters such as the temperature of the particle and the substrate, as well as the particle’s impact velocity, influence not only the extent of deformation but also the predominant bonding mechanism at the interface. A critical velocity exists above which bonding occurs.^1,2,4,12,35^ In polymers, this velocity is lower than, for example, in metals due to their lower yield stress and conductivity.^33^ For the CS deposition of polyolefin microparticles on high density polyethylene, Xu et al. found that the plastic deformation number at the critical velocity of 100 m/s was close to unity.^33^ This roughly matches the plastic deformation number of the here presented system (determined from Figure 5b as described above). Finite element simulations of the CS process of PS particles onto PS substrates show that the global temperature remained below *T*_*G*_, in contrast to the here presented results.^14^ This can be explained by the differences in particle size, with CS particles having a diameter of around 40 μm.

In CS experiments, PS particles on PS substrates showed no adhesion,^14^ in contrast to our experiments, where PS adheres on PS bonds while undergoing viscoplastic flow, possibly due to the observed temperature increase. For CS of PS on silicon substrates, adhesion was observed only at high velocities, with transitions from brittle to viscous flow and jetting.^14^ This hints at a much stronger temperature increase under these CS conditions and confirms that brittle fracture vs ductile transformation observed in CS is related to both materials properties and process parameters (see above).^14^

In our PAD data, no jetting appeared either experimentally or in simulations; unlike in CS of PS on silicon, and many systems involving metals. Jetting in metals is linked to adiabatic shear instability (ASI)^12,35^ but its mechanistic origin remains unclear for polymers. ASI requires localized heating and possibly also pressure release.^13^ In our PAD system of PS on PS, this is probably not observed due to fast thermal energy dissipation (Figure 6e).

## Conclusion

Powder aerosol deposition (PAD) is a solvent-free, room-temperature process that gives access to unique multilayer, multicomponent materials with strong substrate and interfacial adhesion. For these materials, applications in renewable energy production (e.g., dye-sensitized solar cells) or energy storage (e.g., supercapacitors), as coatings for medical devices (e.g., implants), and in microelectromechanical systems including sensors are envisioned.

In this study, we have investigated the deformation and adhesion behavior of polymer particles in the initial stages of PAD using PS on PS substrates as a model system. Atomic force microscopy and molecular dynamics simulations revealed a consistent particle morphology characterized by dome-like cross-sectional and quasi-circular top view shapes. These observations suggest a dominant role of viscoplastic flow in the particles during impact. Despite large differences in particle size and impact energy between experiments and simulations, the overall deformation characteristics were remarkably similar. This consistency is attributed to the universality of key dimensionless numbers such as the plastic number *Pl* = σ_*y*_/σ_*k*_, which govern the flow behavior across scales. This is even true for the larger experimental scales of CS experiments,^33^ when sufficient energy for plastic flow of the CS particles was available. Our results suggest that initial particle adhesion, and possibly also deposition of further particles, in PAD of polymers that have the glass transition temperature *T*_*G*_ in a suitable range is primarily governed by viscoplastic deformation rather than by fragmentation as typically observed in PAD of ceramic systems, or jetting due to adiabatic shear instabilities which are found in CS process under certain conditions. These insights suggest that for efficient PAD of polymers, materials with good plastic deformability, rather than good fragmentation, may be favored. We envision that thermoplastic elastomers, copolymers with rubbery components, and even polymer particles containing softeners will show this kind of adhesion and deposition behavior, and potentially also a better deposition efficiency than polymers with a high *T*_*G*_, or none at all. Our study thus contributes to identifying material properties and design principles for successful polymer PAD. However, in this context care must be taken not to understand the here investigated system as a universally valid blue print for all polymers in PAD. It is well possible that other types of polymer, e.g. those with high *T*_*G*_ or with cross-links, will undergo fracturing, as observed for some polymers in CS, and for ceramics particles in PAD. Some high *T*_*G*_ polymers, e.g., conductive polymers like polypyrrole, polyaniline and polythiophene derivatives, are technologically important, so that future studies should investigate the scope and limits of the here presented principles for polymer PAD for these systems.

## Supplementary Material





## Notes

Snapshots from impact animation of a 14 nm PS particle hitting the substrate at 1,000 m/s (PDF)
Impact animation of a 14 nm PS particle hitting the substrate at 1,000 m/s (GIF)
