# Supplementary material for: High-Velocity Impact of Polymer Aerosol Particles on Soft Substrates: Experiments and Simulations
Source: Langmuir. 2025 Dec 11;41(50):33848–56. doi: 10.1021/acs.langmuir.5c03939 (PMC12751019; doi:10.1021/acs.langmuir.5c03939)
Supplement: Supplementary file 1 [file la5c03939_si_001.pdf]

Supporting Information

High-velocity Impact of Polymer Aerosol Particles on Soft Substrates: Experiments and Simulations

Marc C. Thiel,[†,‖] Hongyu Gao,[‡,‖] Matthias B. B. Brzoska,[†,¶] Lukas Siegwardt,[¶] Markus Gallei,[¶,§] Martin H. Müser,[*,‡] and Karen Lienkamp[*,†,§]

[†] Chair of Polymer Materials, Campus C 4.2, Saarland University, 66123 Saarbrücken, Germany
[‡] Chair of Materials Simulation, Campus C 6.3, Saarland University, 66123 Saarbrücken, Germany
[¶] Chair of Polymer Chemistry, Campus C 4.2, Saarland University, 66123 Saarbrücken, Germany
[§] Saarene, Saarland Center for Energy Materials and Sustainability, Campus C 4.2, 66123 Saarbrücken, Germany
[‖] Contributed equally to this work
[*] Corresponding authors: martin.mueser@mx.uni-saarland.de, karen.lienkamp@uni-saarland.de

Number of pages: 2
Number of figures: 1
Number of schemes: 0
Number of tables: 0

Table of contents:

Figure S1: Snapshots from impact animation of a 14 nm PS particle hitting the substrate at 1000 m/s. (.png)

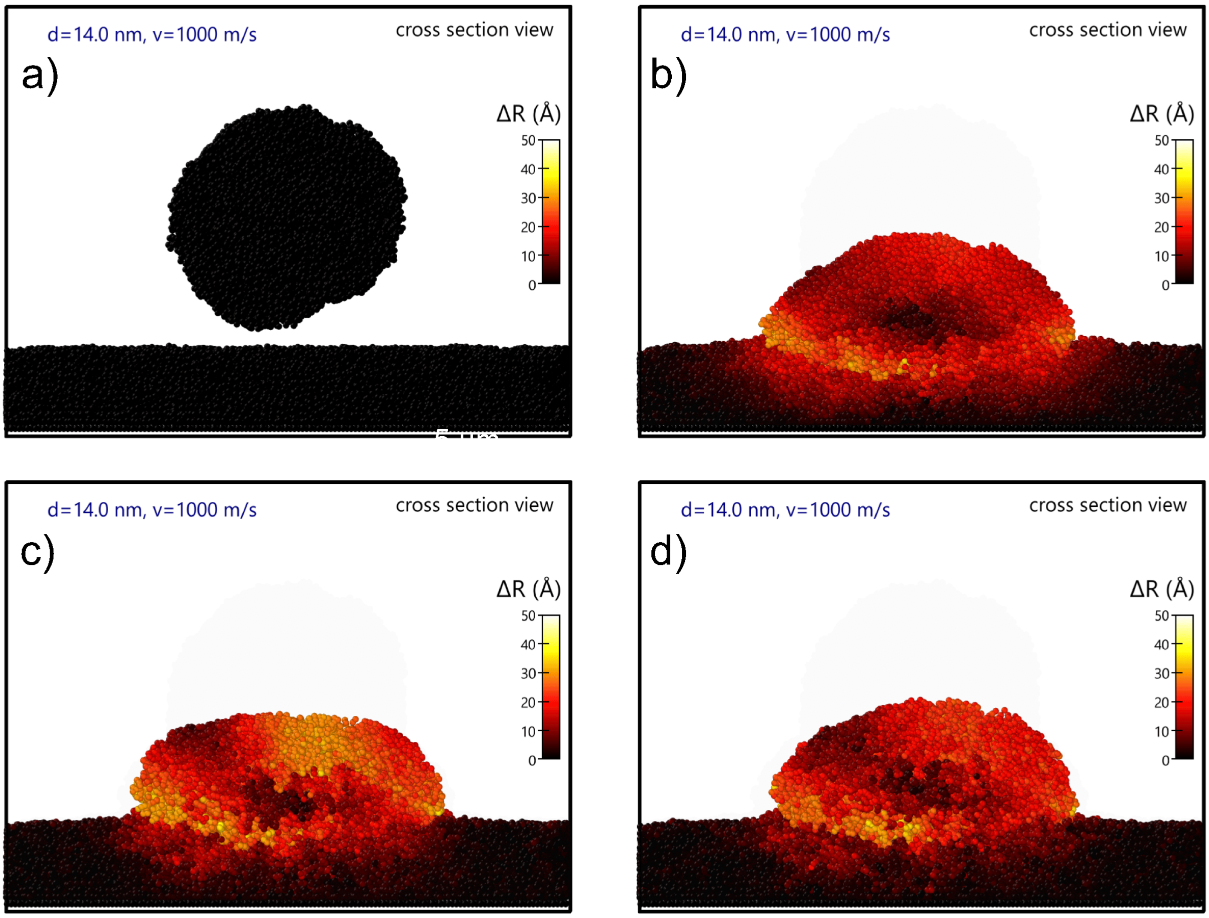

Figure S1: Snapshots from the impact animation of a 14 nm PS particle hitting the substrate at 1000 m/s (see full animation as .gif under Supporting Information).
