# Supplementary figures and images for: High-Velocity Impact of Polymer Aerosol Particles on Soft Substrates: Experiments and Simulations
Source: Langmuir. 2025 Dec 11;41(50):33848–56. doi: 10.1021/acs.langmuir.5c03939 (PMC12751019; doi:10.1021/acs.langmuir.5c03939)

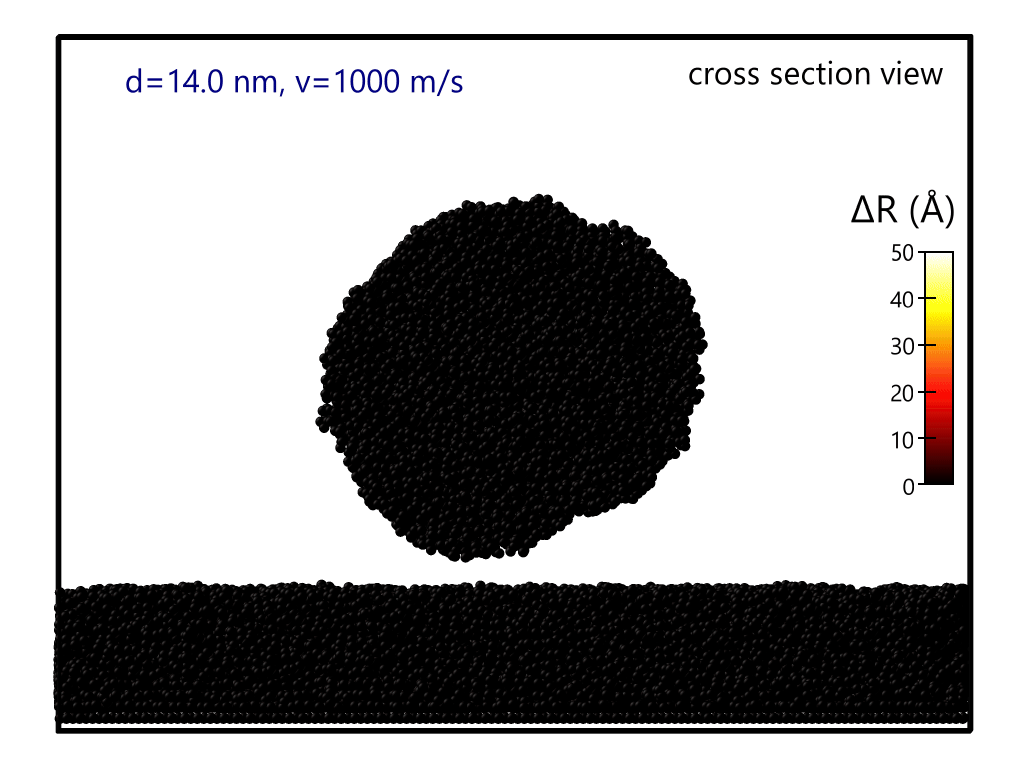

Supplement: Supplementary file 2 [file la5c03939_si_002.gif]
